# Meter-Scale Long Connectorized Paper-like Polymer Waveguide Film for 100 Gbps Board-Level Optical Interconnects Application †

**DOI:** 10.3390/polym16233350

**Published:** 2024-11-29

**Authors:** Xu Liu, Lin Ma, Ying Shi, Qiancheng Yu, Motoya Kaneta, Xu Sun, Zuyuan He

**Affiliations:** 1 State Key Laboratory of Advanced Optical Communication Systems and Networks, Shanghai Jiao Tong University, Shanghai 200240, China; 2 Circuitry with Optical Interconnection Business Development, Sumitomo Bakelite Co., Ltd., 20-7, Kiyohara Kogyo Danchi, Utsunomiya 321-3231, Japan; 3 Crealights Technology Co., Ltd., 1 Pingsheng Road, Suzhou 215126, China

**Keywords:** optical interconnections, optical waveguides, laser direct inscription

## Abstract

We design and fabricate meter-scale long connectorized paper-like flexible multimode polymer waveguide film with a large bandwidth-length product (BLP) for board-level optical interconnects application. The measured BLP of the multimode waveguide is greater than 57.3 GHz·m at a wavelength of 850 nm under the strictest overfilled launch condition with a maximum length of 2.1 m and 10-dB insertion loss. The fabricated waveguide films are as flexible as regular printing paper and can be conveniently interfaced with standard mechanically transferable (MT) fiber connectors with low loss. The average insertion loss of the connectorized waveguide is about 0.042 dB/cm with inter-channel crosstalk as low as −46.4 dB, and the bending loss is less than 1 dB at a bending radius of 1 mm under the overfilled launch condition. We also demonstrate a vertical-cavity surface-emitting laser (VCSEL)-based single-lane 100 Gbps PAM4 transmission. Our results show that the meter-scale long paper-like polymer waveguide film has both excellent optical properties and large bandwidth and is ideal for high-speed board-level optical interconnects application with a single-lane data rate of 100 Gbps and beyond, especially those that have a strict requirement on the length of connection and compactness.

## 1. Introduction

With the development of high-performance computers (HPC) and hyperscale data centers, especially driven by the rapid progress in artificial intelligence (AI) and AI-generated content (AIGC), the demands for computing capability and data interconnects are increasing significantly [1,2,3]. There are increasing demands for high-speed and low-latency board-level and chip-to-chip interconnects. such as all-to-all GPU communications, in order to optimize overall system performance [4,5]. Electrical 100 Gb/s and 112 Gb/s single-lane interconnect standards are defined by IEEE 802.3ck and OIF CEI-112G [6,7].

However, due to the skin effect of copper itself in the printed circuit board (PCB), the loss of copper wires increases significantly with the transmission data rate [8,9]. Optical interconnects have intrinsic advantages in terms of bandwidth, potential in data rate evolution, and power consumption in comparison with their electrical counterparts [10,11]. Active optical devices for board-level optical interconnects with a single-lane 4-level pulse amplitude modulation (PAM4) transmission rate above 100 Gbps have been reported [12,13,14].

Polymer waveguides are attractive transmission media in board-level optical interconnects application due to their advantages such as low transmission loss, large bandwidth, low cost, and mass-producibility [15,16,17,18,19,20,21]. Interconnecting chips and pluggable optical modules with multimode waveguides instead of traditional copper wires can significantly reduce link loss and power consumption [22,23]. In addition, multimode polymer waveguides can be conveniently coupled to and from 50-µm multimode fibers (MMFs) with a negligible coupling loss and a large alignment tolerance [24,25]. Standardized pluggable interfaces offer significant benefits for maintaining optical circuit boards (OCBs) and adjusting their topological networks. OCBs with pluggable interfaces based on multimode polymer waveguides have been reported [26,27]. The flexibility of polymer waveguides also provides obvious advantages in board-level optical interconnects application where space is limited [28,29,30]. Compared to MMF ribbons, flexible polymer waveguide films fabricated using polynorbornene by a “Photo-addressing” technology have a much slimmer thickness and show little degradation in bandwidth due to bending or twisting when they are bent three times at a bending radius of 1 mm or twisted for four full turns, respectively [31]. The meter-scale polymer waveguide can be integrated into larger-area OCBs, facilitating the accommodation of more complex interconnect topologies and more optical chips and devices with ease. A 40 Gbps non-return-to-zero (NRZ) transmission and a 56 Gbps PAM4 transmission in 1 m-long waveguide have been demonstrated [32,33]; however, there are few reports on the meter-scale multimode polymer waveguide that support single-lane 100 Gbps transmission [34].

In this paper, we demonstrated single-lane 100 Gbps VCSEL-based PAM4 transmission at a wavelength of 850 nm over 12-channel meter-scale long connectorized paper-like flexible multimode polymer waveguide films with a large BLP to the best of our knowledge. The measured BLP of the multimode waveguide with a graded index in the horizontal direction is greater than 57.3 GHz·m at 850 nm under the strictest overfilled launch condition with a maximum length of 2.1 m and 10-dB insertion loss. The average insertion loss of the connectorized waveguides is about 0.042 dB/cm with inter-channel crosstalk as low as −46.4 dB under the overfilled launch condition. The flexible polymer waveguide has lower bending loss compared to that of the OM3 MMF ribbon when bent to a radius of less than 6 mm. The measured polarization-dependent loss (PDL) of both 1 m and 1.7 m-long waveguides is not larger than 0.05 dB under both the underfilled and overfilled conditions. The flexible polymer waveguide exhibited a loss increase of less than 0.5 dB following 2000 h of a reliability test under conditions of 85% relative humidity and a temperature of 85 °C. The experimental results show that the meter-scale long paper-like polymer waveguide film has both excellent optical properties and large bandwidth and is suitable for high-speed board-level optical interconnects application with a single-lane data rate of 100 Gbps and beyond, especially those that have a strict requirement on the length of connection and compactness.

## 2. Waveguide Fabrication

**Figure 1:**
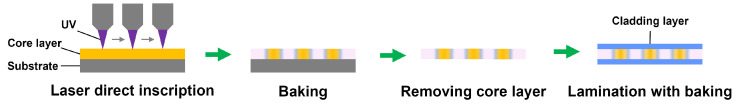
The fabrication process of the flexible polymer waveguide.

The flexible polymer waveguides were fabricated using polynorbornene by the “photo-addressing” technology proposed by Sumitomo Bakelite Co., Ltd. (Tokyo, Japan) [35]. The fabrication process is depicted in Figure 1. First, the material of the core layer is coated onto the substrate, followed by a laser direct inscription (LDI) process for waveguide patterning. The LDI process allows for uniform exposure over a large area without being limited by the size of the mask. Then, the core layer is removed from the substrate after a baking process and sandwiched between upper and lower cladding layers and this process is also finished with baking. Last, a 25 µm-thick polyimide film was laminated on both sides of the waveguide for protection. No wet development is needed throughout the fabrication processes, which effectively minimizes the sidewall roughness of the core and makes the fabrication process more environmentally friendly. Without the detrimental effect of sidewall roughness, the propagation loss of the waveguide can be significantly optimized [19].

**Figure 2:**
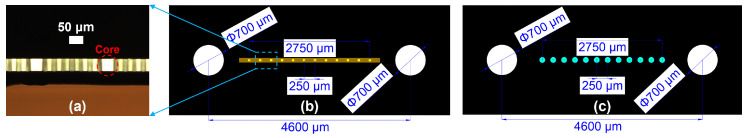
(**a**) Microscope image of the fabricated polymer waveguides, (**b**) 12-core MT connector for polymer waveguides, and (**c**) 12-core MT connector for OM3 optical fibers.

The refractive index of the core is about 1.55 at a wavelength of 850 nm, and the refractive index of the horizontal and vertical cladding is 1.53 and 1.52, respectively [36]. As shown in Figure 2, the core size of the waveguide is about 43 × 44 µm and each waveguide group contains single-row 12-channel waveguides with a core pitch of 250 µm, which coincides with that of the commercially available VCSELs, photodiode arrays, and MMF ribbons. The waveguides are connectorized using single-row 12-channel polymer mechanically transferable (PMT) connectors in accordance with the recommendation of IEC 62496-4-1 (Figure 2b), which guarantees a low-loss interconnection with 12-channel fiber MT connectors in accordance with IEC 61754-5 (Figure 2c).

**Figure 3:**
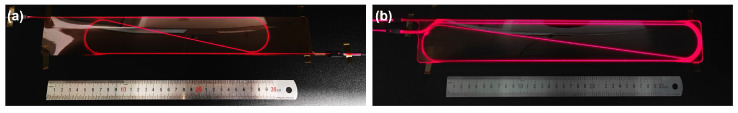
Images of (**a**) 1 m long and (**b**) 1.7 m long polymer waveguide illuminated by a red identification light.

**Figure 4:**
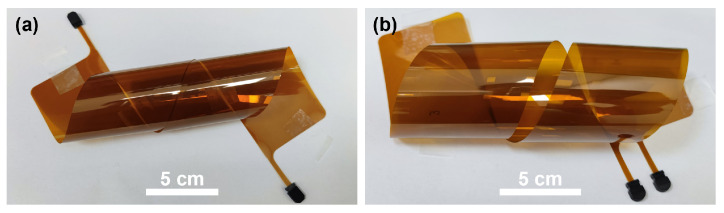
(**a**) 1 m long and (**b**) 1.7 m long polymer waveguide films under twisting conditions.

The fabricated 1 m and 1.7 m long waveguide films illuminated by a red identification light are shown in Figure 3. Both have a length of about 43.5 cm and a width of 5.5 cm. The thickness of them with the polyimide protection films is about 110 µm, which is equivalent to that of a sheet of printing paper and is over 60% thinner than the MMF ribbon, which usually has a thickness of about 300 µm. Figure 4 demonstrates the flexibility of the fabricated 1 m long and 1.7 m long waveguide film, respectively. In order to minimize the in-plane bending loss and make full use of the area of the waveguide film, the minimum in-plane bending radius of the 1 m long and 1.7 m long waveguides is designed to be 24 mm and 21 mm, respectively.

## 3. Optical Characteristics

**Figure 5:**
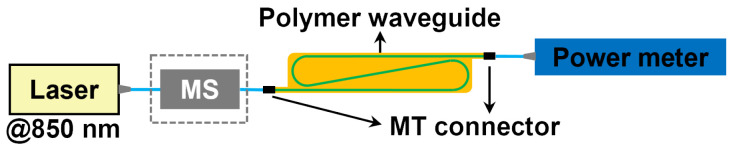
Experimental setup for the insertion loss measurement.

The insertion losses of the 12-channel connectorized waveguides under overfilled and underfilled launch conditions at a wavelength of 850 nm were measured with and without the use of a mode scrambler (MS, Newport’s FM-1), respectively. A schematic of the measurement setup is shown in Figure 5.

**Figure 6:**
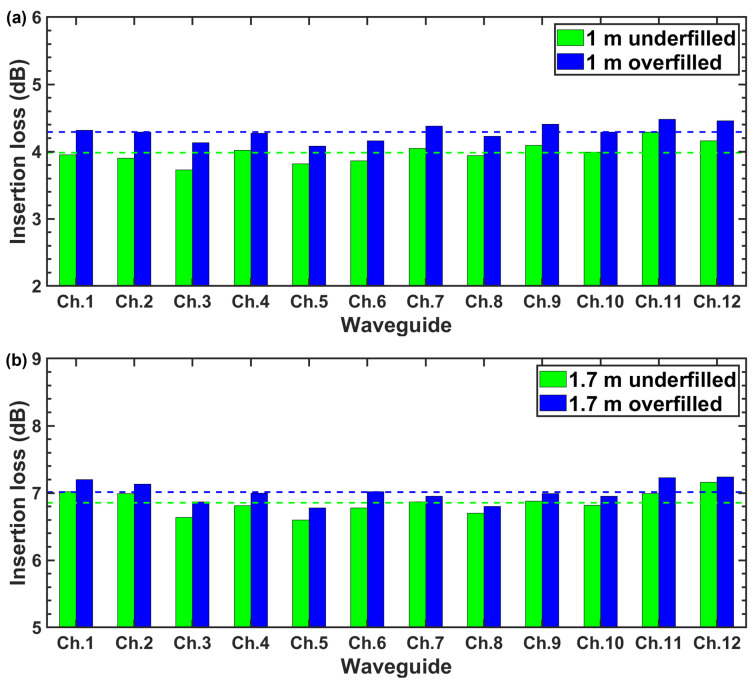
(**a**) Insertion loss of 1 m long waveguide and (**b**) insertion loss of 1.7 m long waveguide.

The averaged 12-channel insertion loss of the 1 m-long waveguides under overfilled and underfilled launch conditions is 4.3 dB (0.043 dB/cm) and 4.0 dB (0.040 dB/cm), respectively, as shown in Figure 6a. The averaged 12-channel insertion loss of the connectorized waveguides with a length of 1.7 m under overfilled and underfilled launch conditions is 7.0 dB (0.042 dB/cm) and 6.9 dB (0.040 dB/cm), respectively, as shown in Figure 6b. The additional loss due to the increased number of curves and adoption of a tighter bending radius for a 1.7 m long waveguide sheet is negligible. Moreover, the slightly higher loss of about 0.002 to 0.003 dB/cm under the overfilled launch condition implies that a larger portion of the light transmits in higher order modes, which have a higher bending loss in comparison with that of the underfilled launch condition.

**Figure 7:**
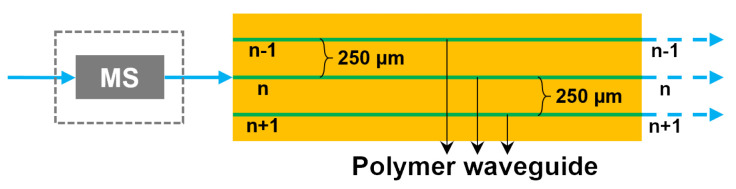
Schematic of the inter-channel crosstalk measurement.

The inter-channel crosstalk of the fabricated 12-channel polymer waveguides was measured using the setup as shown in Figure 7. When laser light operating at 850 nm is input from the waveguide numbered n, its inter-channel crosstalk can be obtained by measuring the received optical power at the outputs of the adjacent waveguides numbered n − 1, n, and n + 1. The measurement results of inter-channel crosstalk are shown in Table 1. The maximum crosstalk under the strictest overfilled launch working condition is as low as −46.4 dB for the 1.7 m long waveguide film.

**Figure 8:**
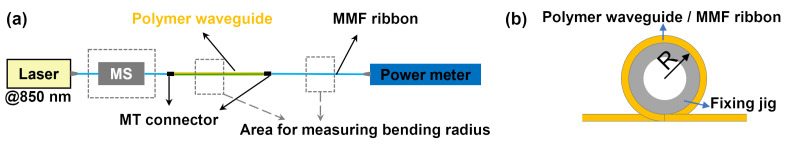
(**a**) System setup for measuring the bending loss and (**b**) schematic of a polymer waveguide/MMF being bent into a full circle.

The experimental setup for measuring the bending loss of flexible waveguide film and MMF ribbon is shown in Figure 8a. The polymer waveguide film and MMF ribbon in the same link are bent into a full circle using fixing jigs with various radii, as shown in Figure 8b.

**Figure 9:**
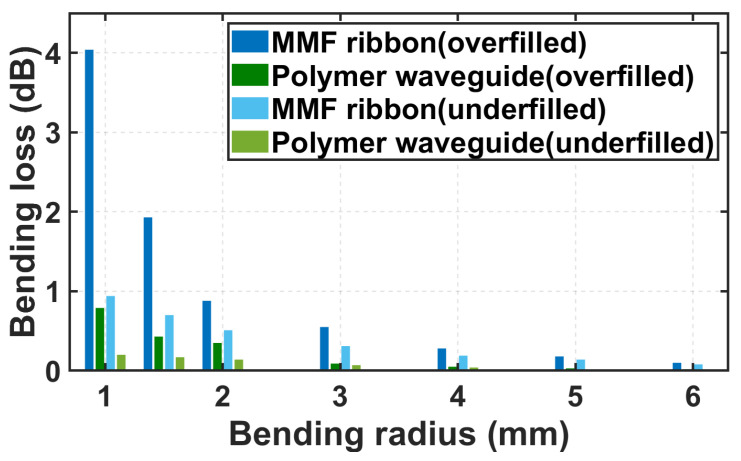
Comparison of the bending loss in the polymer waveguide and the MMF ribbon.

The measured bending loss under both the overfilled and underfilled launch conditions is shown in Figure 9. The fabricated polymer waveguide films exhibit a much smaller bending loss than that of the MMF ribbon with an additional loss of less than 1 dB at a bending radius as small as 1 mm under the overfilled launch condition. In addition, the mechanical flexibility of the fabricated polymer waveguide films is superior to that of the MMF ribbon due to both their thinner film thickness and the nature of polynorbornene materials over silica glass. Therefore, our polymer waveguide film presents significant advantages for applications with tight spatial constraints, such as interconnection of miniature on-board transceivers.

**Figure 10:**
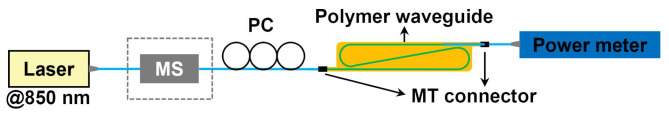
System setup for measuring the polarization-dependent loss.

The PDL of the flexible waveguide under both the underfilled and overfilled conditions was measured using the experimental setup shown in Figure 10. Table 2 shows that the measured PDL of both 1 m long and 1.7 m-long waveguides was not higher than 0.05 dB under both of the launch conditions.

The flexible polymer waveguide exhibited a loss increase of less than 0.5 dB following 2000 h of the reliability test under conditions of 85% relative humidity and a temperature of 85 °C. The test results demonstrate that the waveguide exhibits excellent thermal reliability.

## 4. Bandwidth Study

**Figure 11:**
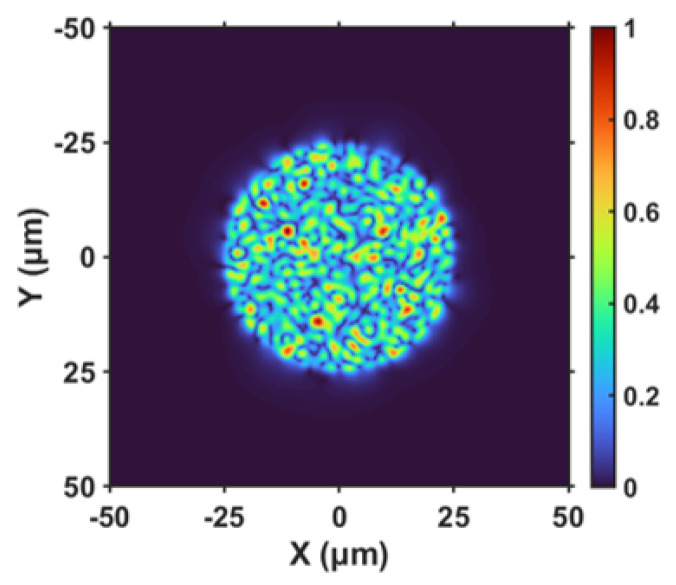
Optical field input by 50-µm MMF.

First, we carried out the theoretical study on the bandwidth of the polymer waveguide using a beam propagation method (BPM) and the refractive index distribution of the calculation model is the same as that of the fabricated ones. In order to simulate the overfilled launch condition to excite as many modes as possible in the polymer waveguide, we chose an optical field of 50 µm-core MMF with a random distribution of modes as the input optical field, as shown in Figure 11.

**Figure 12:**
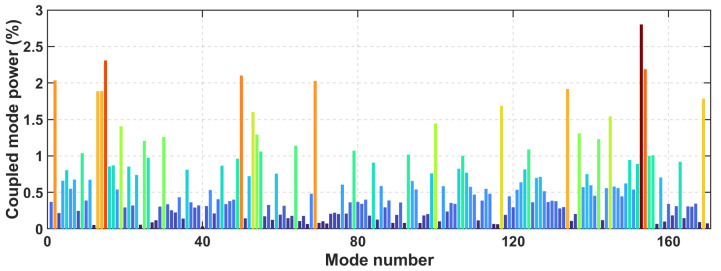
Normalized mode power distribution of the waveguide under the simulated overfilled launch condition.

At a wavelength of 850 nm, altogether, 170 modes of the waveguide with a normalized power percentage higher than 1% of that of the mode with the highest power can be excited, and their power distribution is shown in Figure 12.

The group refractive indices of these modes can be calculated by

(1) $$n_{g}=n_{eff}-\lambda \frac{\partial n_{eff}}{\partial \lambda }$$

where $n_{eff}$ is the effective refractive index of the mode, and $n_{g}$ is its group refractive index [37].

The differential mode delay (DMD) of the waveguide under the overfilled launch condition is defined by the difference between the maximum and minimum values of the group refractive indices [38], which can be calculated by

(2) $$DMD=\max\left(n_{g}\right)-\min\left(n_{g}\right)$$

The calculated BLP of the polymer waveguide under the strictest overfilled launch condition is about 52.4 GHz·m, which is the reciprocal of DMD. Under the same simulation conditions, the BLP of the waveguide with a horizontal refractive index gradient is larger than that of the waveguide with a step refractive index (48.4 GHz·m) of the same size.

Although we successfully measured the BLP of the polymer waveguides by connecting 20 cm long polymer waveguides into a meter-scale long waveguide link using a unique optical sampling method, the implementation of many polymer waveguide connectors will inevitably result in bandwidth degradation due to the misalignment at the interface [31]. However, it is challenging to characterize the BLP of the waveguide using well-established measurement technology of $S_{21}$ parameters due to the short length of the waveguide and the limited bandwidth of the vector network analyzer.

**Figure 13:**
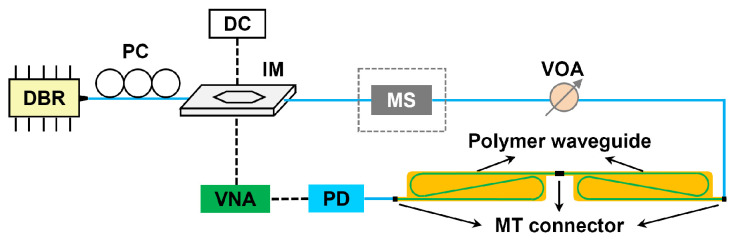
Experimental setup for the $S_{21}$ response measurement.

In order to directly evaluate the BLP of fabricated waveguides, a vector network analyzer (VNA), was used to measure the $S_{21}$ response curves of waveguide links. A schematic of the experimental setup is shown in Figure 13. We obtained waveguide links with different lengths by connecting waveguides with a length of 0.2 m, 1 m, and 1.7 m, respectively. The light from a distributed Bragg reflection (DBR) laser operating at 850 nm passed through a polarization controller (PC) and entered an intensity modulator (IM) to be modulated. The modulated light was then injected into a multimode variable optical attenuator (VOA) and launched into the waveguide through a 50 µm-core OM3 multimode fiber.

**Figure 14:**
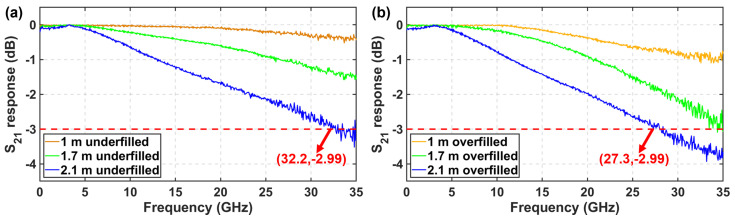
(**a**) Normalized $S_{21}$ response curves under the underfilled launch condition and (**b**) the overfilled launch condition.

The normalized $S_{21}$ response curves measured under underfilled launch conditions are shown in Figure 14a. The attenuation of the $S_{21}$ response curve at 32.2 GHz is less than 3 dB for a waveguide link with a length of 2.1 m and an insertion loss of 10 dB. As a result, the BLP of the waveguide under the underfilled launch condition is greater than 67.6 GHz·m. The normalized $S_{21}$ response curves measured under the overfilled launch condition are shown in Figure 14b. The attenuation of the $S_{21}$ response curve at 27.3 GHz is less than 3 dB for a waveguide link with a splice length of 2.1 m. As a result, the BLP of the waveguide under the overfilled launch condition is greater than 57.3 GHz·m. The measured result agreed well with the simulated ones.

The measured BLP of the waveguide link spliced by 20 cm-long polymer waveguides fabricated by the same process at a wavelength of 780 nm is about 42 GHz·m using an optical sampling technique [31]. The difference between the two results may result in both a large reduction in the number of connections in the waveguide link and the difference in wavelength.

## 5. Transmission Experiment

**Figure 15:**
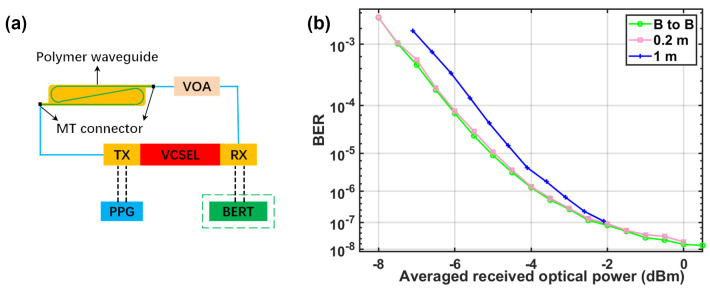
(**a**) Experimental setup for 53.125 Gbaud PAM4 transmission and (**b**) BER curves for the B2B and waveguide links with different lengths.

Single-lane 100 Gbps PAM4 VCSEL-based transmission over the fabricated 1 m long polymer waveguides was carried out. A schematic of the experimental setup is shown in Figure 15a. The transceiver (TX) of the VCSEL received the radio frequency signal of 53.125 Gbaud PAM4 from the pulse pattern generator (PPG) and output the modulated light at 850 nm. The modulated light passes through the waveguide and is connected to the multimode VOA. The receiver (RX) of the VCSEL received the modulated light and passed the corresponding RF signal into the bit error ratio tester (BERT) to obtain the bit error rate (BER) of the link. Figure 15b shows measured BER curves of 53.125 Gbaud PAM4 signal versus different received optical powers for the B2B link and waveguide links with various lengths. The measured BERs are well below the forward error correction limitation (BER = 3.8 × $10^{-3}$). Compared with the B2B link, the signal after 1 m long waveguide transmission suffers a power penalty of less than 1 dB.

**Figure 16:**
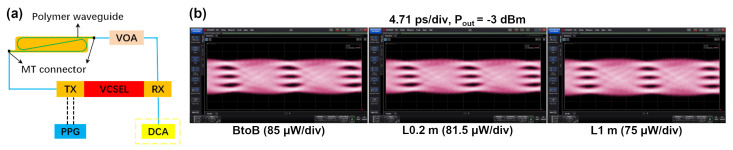
(**a**) Experimental setup and (**b**) received eye diagrams for the B2B and waveguide links with different lengths.

Eye diagrams of 100 Gbps PAM4 VCSEL-based transmission were obtained using a digital communications analyzer (DCA). A schematic of the experimental setup is shown in Figure 16a. Figure 16b shows the 100 Gbps PAM4 eye diagrams for the B2B link and waveguide links with different lengths when the received optical power is −3 dBm, respectively. The eye diagrams open well after the propagation through a 1 m-long waveguide and the transmitter dispersion eye closure penalty quaternary (TDECQ) of the eye diagram is about 3 dB. The measurement standard for TDECQ is IEEE 802.3cd [39] and the target symbol error rate (SER) is set to 4.8 × $10^{-4}$.

## 6. Conclusions

In conclusion, we successfully demonstrated a connectorized meter-scale paper-like flexible multimode polymer waveguide film that supports single-lane 100 Gbps PAM4 VCSEL-based transmission for board-level optical interconnects application. The measured BLP of the multimode waveguide is greater than 57.3 GHz·m at 850 nm under the strictest overfilled launch condition with a maximum length of 2.1 m and 10 dB insertion loss. The waveguides are as flexible as regular printing paper and can be conveniently interfaced with standard MT fiber connectors with low loss. The connectorized waveguide has an average insertion loss of approximately 0.042 dB/cm. Furthermore, the inter-channel crosstalk is as low as −46.4 dB under the overfilled launch condition. The bending loss of the polymer waveguide is less than 1 dB at a bending radius of 1 mm. The polymer waveguide has low polarization sensitivity and good thermal stability. These results show that the meter-scale long paper-like polymer waveguide film has both excellent optical properties and large bandwidth and is suitable for multimode high-speed board-level optical interconnects application with a single-lane data rate of 100 Gbps and beyond.

## Figures and Tables

**Table 1:** Measurement results of inter-channel crosstalk.

Length	Underfilled	Overfilled
1 m	≤−50.8 dB	≤−48.0 dB
1.7 m	≤−48.5 dB	≤−46.4 dB

**Table 2:** Measurement results of the polarization-dependent loss of the polymer waveguides.

Length	Underfilled	Overfilled
1 m	≤0.03 dB	≤0.05 dB
1.7 m	≤0.05 dB	≤0.05 dB

## Data Availability

The raw data supporting the conclusions of this article will be made available by the authors on request.
